# Construction of a Gradient Nanostructure for Enhanced Surface Properties in 38CrMoAl Steel via Ultrasonic Severe Surface Rolling

**DOI:** 10.3390/ma18235308

**Published:** 2025-11-25

**Authors:** Jing Han, Yongzheng Zha, Tao Zhang, Haiyong Shi, Xingyue Zhang, Chao Cao, Di Huang, Jiapeng Sun, Bin Zhang, Jiyun Zhao

**Affiliations:** 1 School of Mechanical and Electrical Engineering, China University of Mining and Technology, Xuzhou 221116, Chinacaoch@cumt.edu.cn (C.C.);; 2 State Key Laboratory of Intelligent Mining Equipment Technology, China University of Mining and Technology, Xuzhou 221116, China; 3 State Key Laboratory of Fluid Power and Mechatronic Systems, Zhejiang University, Hangzhou 310027, China; zbzju@163.com; 4 Suzhou Liyuan Hydraulic Co., Ltd., Suzhou 215143, China; 5 College of Materials Science and Engineering, Hohai University, Nanjing 210024, China

**Keywords:** gradient, 38CrMoAl, tempering, strength, wear

## Abstract

Fabrication of gradient nanostructure on metal surfaces is recognized as an effective approach for enhancing mechanical and surface properties, as well as serving as a pretreatment for subsequent surface engineering. Unfortunately, their fabrication on high-strength and low-ductility metal surface poses a significant challenge due to the prevalent issue of process-induced surface damage. In this study, we report the successful fabrication of a gradient nanostructured surface layer with low roughness (Ra ~ 0.17 μm) on high-strength 38CrMoAl steel through an optimized ultrasonic severe surface rolling (USSR) processing. By systematically varying the tempering temperature of quenched-and-tempered samples, the strength and ductility of the 38CoMoAl steel are tailored to facilitate gradient nanostructure formation. Microstructural analysis via advanced electron microscopy reveals the gradient nanostructure features progressively coarser martensite/ferrite grains and decreasing dislocation density along the depth. As the tempering temperature increases from 600 °C to 700 °C, the yield strength of 38CrMoAl steel decreases from 915 ± 16 MPa to 815 ± 16 MPa, while the elongation increases from 18.7 ± 0.6 to 27.3 ± 1.2%, resulting in an increase in the thickness of the gradient nanostructured surface layer from 300 μm to 400 μm. Following USSR processing, samples tempered at 600 °C, 650 °C, and 700 °C exhibit significant enhancements in surface hardness by 7.3%, 22.7%, and 21.5%, respectively, along with substantial reduction in wear volume by 73%, 78%, and 60%. USSR processing also leads to a reduction in coefficient of friction. This work provides valuable insights into the fabrication of high-quality gradient nanostructures on high-strength, low-ductility metallic materials.

## 1. Introduction

Gradient nanostructures are defined by a smooth, spatially continuous variation in microstructural features—such as grain size, twin density, and phase composition—from the material’s surface to its interior [1,2,3]. Unlike homogeneous materials, this microstructural architecture emulates the sophisticated gradation found in natural systems like bamboo. The key advantage of this nanostructure lies in achieving a superior combination of multiple properties that are often mutually exclusive in conventional materials. The nanograined surface layer provides exceptionally high strength and hardness, significantly enhancing wear resistance and fatigue life. Meanwhile, the coarser-grained core maintains good ductility and toughness, effectively arresting cracks that might initiate at the surface. This harmonious synergy between surface strength and core toughness circumvents the common strength-ductility trade-off. Consequently, these unique characteristics render gradient nanostructured materials viable candidates for demanding applications in sectors such as construction machinery, aerospace, biomedical implants, and the automotive industry, thereby paving the way for next-generation materials with tailored, multifunctional performance.

The past two decades have witnessed considerable research interest in the fabrication of gradient nanostructures, driven by their exceptional combination of properties. Among the various fabrication strategies, surface severe plastic deformation techniques have proven particularly effective. These methods engineer a gradient nanostructure by introducing a gradient strain and strain rate that decreases with depth, leading to a corresponding refinement of grain size from the surface nanometers into the micron-scale coarse-grained interior. A number of surface severe plastic deformation techniques have been developed, including surface mechanical attrition treatment (SMAT) [4], surface mechanical grinding treatment (SMGT) [5], surface mechanical rolling treatment (SMRT) [6,7], ultrasonic surface rolling (USR) [8,9,10], friction stir processing (FSP) [11,12], and laser shock peening (LSP) [13,14,15]. SMAT is a versatile and widely adopted process where the sample surface is bombarded by flying balls, generating random impacts that produce a homogeneous nanograined surface layer. SMGT/SMRT utilize a tool tip or roller that moves relative to the workpiece, imposing a continuous, large strain. LSP employs high-intensity laser pulses to generate plasma-induced shock waves that impart deep compressive residual stresses and a gradient structure with minimal thermal impact. USR combines static pressure with ultrasonic-frequency vibrations, resulting in deep plastic deformation and significant work hardening with exceptionally low surface roughness. A novel technique, ultrasonic severe surface rolling (USSR), has recently been established by our group for fabrication of gradient nanostructures [16,17,18,19]. It imposes severe plastic deformation on the surface layer by synergistically applying a high static load and ultrasonic vibration, consequently forming a gradient nanostructure with significant depth.

Nevertheless, the significant plastic strain introduced by surface severe plastic deformation techniques can readily generate surface defects such as micro-cracks. These surface defects invariably lead to the degradation of surface properties. Hao et al. [20] reported that SMAT processing of 316L stainless steel resulted in a gradient nanostructure featuring 19 nm surface grains, but at the expense of generating extensive surface cracks. This defect formation led to a drastic loss of corrosion resistance, manifested by the disappearance of the passive region in polarization measurements. Furthermore, high surface roughness and cracks predispose the material to fatigue crack initiation, leading to a significant reduction in fatigue life [21]. Minimizing surface plastic deformation enhances surface integrity, but overly conservative processing compromises the gradient layer thickness and may preclude nanograined formation at the surface. This creates a critical processing dilemma, underscoring why fabricating thick, defect-free gradient nanostructures with high surface quality remains a formidable challenge. In fact, most successfully demonstrated gradient nanostructures to date have been produced in metallic materials with good intrinsic ductility, such as 316L stainless steel and copper alloys [1]. The achievement of such structures in high-strength, low-ductility materials continue to pose considerable difficulty.

38CrMoAl (GB/T3077-2015 standard, China) is a classic nitriding alloy steel specifically designed to achieve exceptional surface hardness and wear resistance through nitriding [22]. It is widely used in engineering machinery, such as plungers in high-pressure piston pumps, among other applications. This steel also offers good comprehensive mechanical properties in its quenched and tempered condition. This makes it a suitable material for structural components demanding a combination of high core strength and toughness, even when the exceptional surface properties of nitriding are not the primary requirement. The introduction of a gradient nanostructure on 38CrMoAl steel significantly enhances its surface properties, such as hardness, wear resistance, and the strength-ductility balance. Moreover, this nanostructured layer markedly enhances the diffusion kinetics of nitrogen atoms during nitriding. This catalytic effect promotes a more efficient process, with the added benefit of reducing the required nitriding temperature and time [23,24,25]. By applying SMAT to 38CrMoAl steel, Tong Et Al. produced a gradient nanostructured surface layer, which enabled nitriding to be achieved at a temperature as low as 400 °C [26]. 38CrMoAl steel is typically used in a quenched and tempered condition. Its mechanical properties, which are highly dependent on the tempering temperature, significantly influence the fabrication of the gradient nanostructured surface layer. However, the underlying relationship remains unclear. In fact, producing a defect-free gradient nanostructure on this steel remains a considerable challenge.

This study aims to fabricate a gradient nanostructured surface layer on high-strength 38CrMoAl steel Via a combined quenching–tempering–USSR route, with a focus on elucidating the critical role of tempering temperature. The resulting material exhibits significantly enhanced wear resistance, the underlying mechanism of which is thoroughly analyzed. The findings provide valuable insights for fabricating high-quality gradient nanostructures on high-strength, low-ductility metallic materials.

## 2. Materials and Methods

The commercial 38CrMoAl alloy was subjected to quenching and tempering heat treatment. First, it was held at 940 °C for 2 h and oil-quenched, followed by tempering heat treatment at 600 °C, 650 °C, and 700 °C for 2 h. The resulting samples were labeled as T600, T650, and T700. Subsequently, the quenched and tempered specimens were processed by USSR. The quenched and tempered samples were then processed by USSR. Due to the low plasticity of 38CrMoAl alloy, the severe surface deformation induced by USSR can readily cause surface defects such as cracks and pits. Through experimental optimization, a set of USSR parameters were identified to produce a damage-free surface: a static load of 800 N, an interval of 0.1 mm, a scanning speed of 10 mm/s, and 10 passes.

The microstructure of the quenched and tempered sample was characterized using electron backscatter diffraction (EBSD; Oxford C-nano, OXFORD instruments, Oxford, UK) and transmission electron microscopy (TEM; FEI Talos F200X, Thermo Fisher Scientific, Waltham, MA, USA). For EBSD analysis, the samples were mechanically ground and polished, followed by final polishing with an ion beam. For TEM analysis, the samples were first sectioned into ~500-μm-thick slices using a low-speed saw, mechanically ground to a thin foil, and then thinned by ion milling (PIPS II, Gatan 695, Gatan, Inc., Pleasanton, CA, USA). The microstructure of the USSR-processed samples was characterized on the cross-section using EBSD and optical microscopy (OM, AXIO Lab.A1, ZEISS, Auberkheim, Germany). The EBSD samples were prepared using the same method as the quenched and tempered samples. The OM samples were mechanically ground and polished, followed by chemical etching. The collected EBSD data were processed with the Channel 5 software (v5.0.9.0), from which inverse pole figure (IPF) maps and kernel average misorientation (KAM) maps were obtained.

Room temperature tensile tests were conducted using a universal testing machine (SUNS UTM4204X, Shenzhen, China) with a strain rate of 0.0001 s^−1^. The tensile samples were machined to a gauge length of 7.5 mm with a cross-section of approximately 2 mm × 2 mm. The reproducibility of the data was affirmed by conducting three tests per sample. Vickers microhardness measurement was conducted using an HV-1000 tester (Najing times testing equipment Co., Ltd., Nanjing, China) with a load of 2.94 N, following the ASTM E92-17 standard [27]. Each reported hardness value represented the average of three individual measurements.

Linear reciprocating sliding tribological test was performed using an MS-M9000 multifunctional tribometer (Lanzhou Huahui Instrument Technology Co., Ltd., Lanzhou, China) in a ball-on-flat plate configuration in accordance with ASTM G133-05 [28]. Al_2_O_3_ balls with a diameter of 6 mm were used as the counterpart. A sliding stroke of ~3 mm, a frequency of 5 Hz, a normal load of 2 N, and a duration time of 40 min were chosen. All the wear tests were conducted at room temperature under dry sliding conditions. After the wear test, the width of the wear scar (*W*) was measured using OM. For accuracy, *W* of each wear scar was measured at a minimum of two representative locations, and the mean value was reported. The average of these measurements was then used as the final width. The wear volume (*V*) was subsequently calculated using the formula:

(1) $$V=L\left(\arcsin\left(\frac{W}{2D}\right)R^{2}-0.5W\sqrt{R^{2}-0.25W^{2}}\right)+\pi ({R-\sqrt{R^{2}-0.25W^{2}})}^{2}(R-\frac{R-\sqrt{R^{2}-0.25W^{2}}}{3})$$

where *R* is the radius of the used ball, and *L* is the sliding stoke. Data are expressed as the mean ± standard deviation.

## 3. Results and Discussions

### 3.1. The Influence of Tempering Temperature on the Microstructure and Mechanical Properties

**Figure 1:**
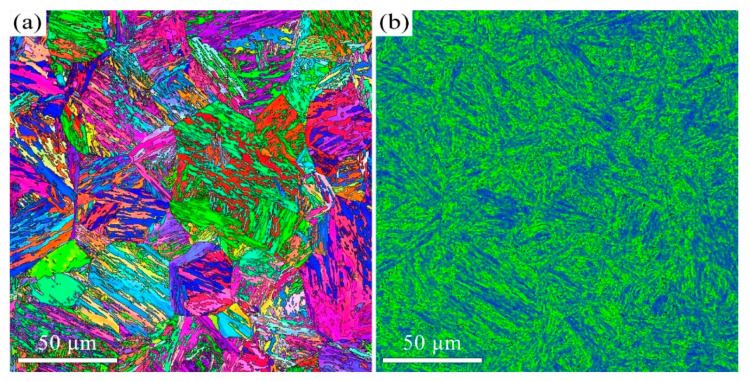
EBSD results of the T600 sample: (**a**) IPF mapping and (**b**) KAM mapping.

**Figure 2:**
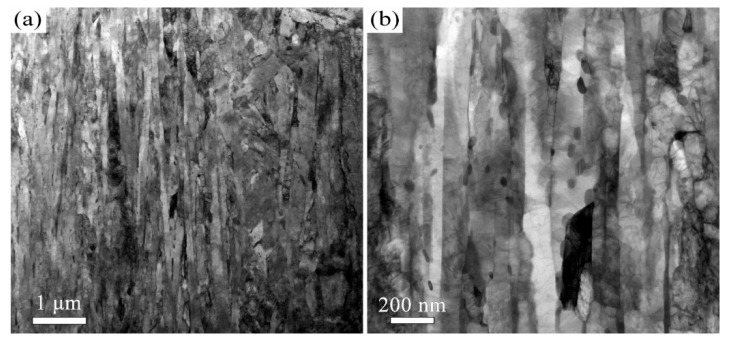
TEM micrographs of the T600 sample: (**a**) Low- and (**b**) high-magnification images.

**Figure 3:**
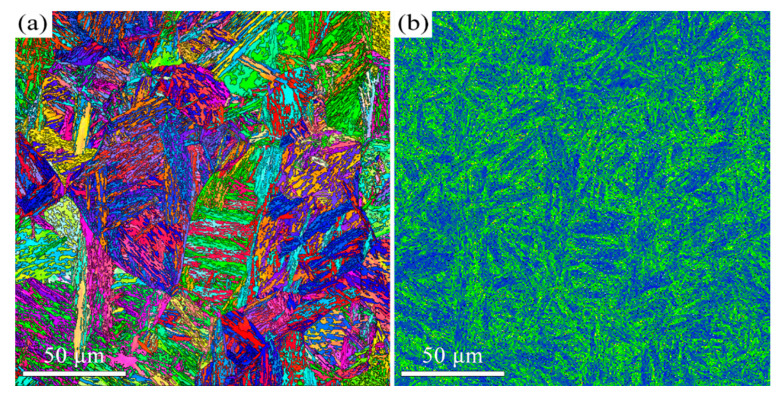
EBSD results of the T650 sample: (**a**) IPF mapping and (**b**) KAM mapping.

**Figure 4:**
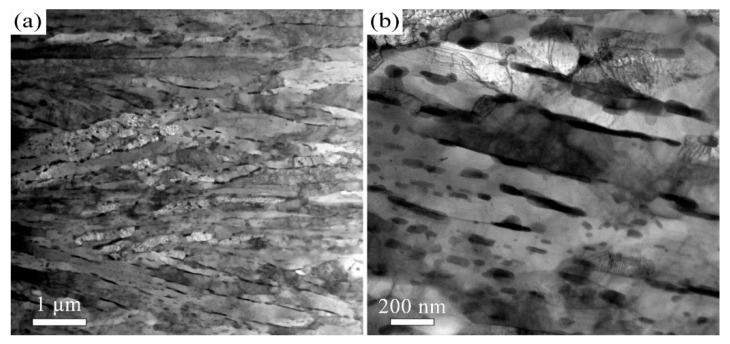
TEM micrographs of the T650 sample: (**a**) Low- and (**b**) high-magnification images.

**Figure 5:**
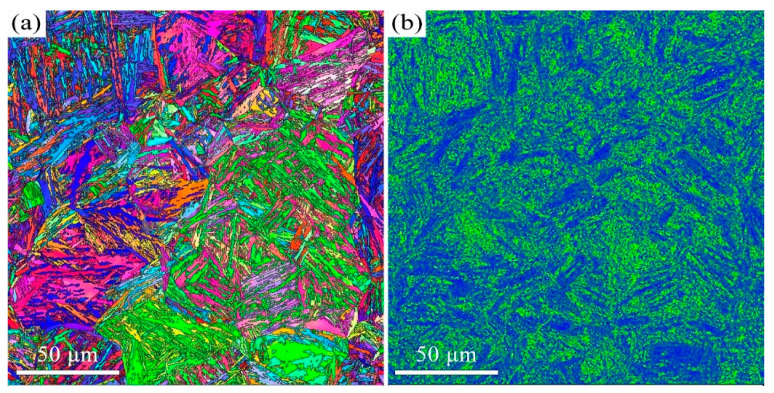
EBSD results of the T700 sample: (**a**) IPF mapping and (**b**) KAM mapping.

**Figure 6:**
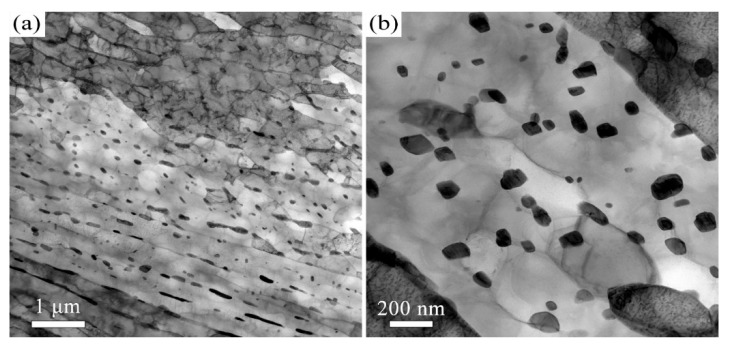
TEM micrographs of the T700 sample: (**a**) Low- and (**b**) high-magnification images.

The quenched microstructure of 38CrMoAl steel is characterized by low-carbon lath martensite. The subsequent tempered microstructure is a function of the tempering temperature. We employed EBSD and TEM to investigate the microstructure of 38CrMoAl steel after tempering at 600 °C, 650 °C, and 700 °C (Figure 1, Figure 2, Figure 3, Figure 4, Figure 5 and Figure 6). After tempering at 600 °C, the lath martensite morphology remains clearly visible, as shown in Figure 1a. The parent austenite grains are hierarchically subdivided into packets, blocks, subblocks, and laths at different microstructural scales. There is no evidence of retained austenite. The T600 sample also exhibits very high KAM value (Figure 1b), averaging up to 1.1°. Since KAM reflects the density of geometrically necessary dislocations (GNDs), the high KAM indicates high GND density. In addition, a small number of nano-scale carbides with an average size of 52 ± 20 nm are observed, as shown in Figure 2. This average size is determined Via image analysis using ImageJ software (v1.54p) and represents the Feret diameter. They are primarily spherical in morphology and are located along the martensite lath boundaries. These results indicate that the decomposition of martensite is limited after tempering at 600 °C.

When the tempering temperature is increased to 650 °C, the martensite morphology becomes increasingly indistinct, as shown in Figure 3 and Figure 4. The average KAM value decreases to 1° (Figure 3b), which corresponds to a reduction in dislocation density. The average carbide size is measured to be 64 ± 32 nm. The Kolmogorov–Smirnov test reveals that this size did not differ significantly from that of the T600 sample. When the temperature is further increased to 700 °C, the features of martensite become barely discernible, as indicated in Figure 5 and Figure 6. The carbides undergo coarsening, reaching an average size of 82 ± 32 nm (Figure 6). The Kolmogorov–Smirnov test demonstrates a statistically significant difference in size from both the T600 and T650 samples. This process is accompanied by a simultaneous increase in their population. A marked reduction in dislocation density is also manifested by the reduced average KAM value of 0.88° (Figure 5b). This suggests that the martensite has fully decomposed into ferrite and carbides. Additionally, the presence of equiaxed ferrite grains is observed, indicating that the spheroidization of ferrite had commenced.

**Figure 7:**
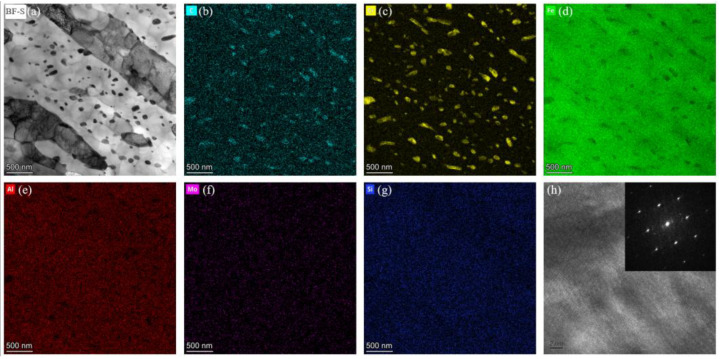
TEM micrographs (**a**) and corresponding element mappings (**b**–**g**) of the T700 sample, (**h**) HRTEM image and FFT pattern of a typical carbide.

To further investigate the structure and composition of the precipitates, we performed additional characterization on the precipitates in the sample tempered at 700 °C. Figure 7 shows the typical TEM morphology and the corresponding elemental distribution of the 700 °C tempered sample. The results reveal significant Cr enrichment within the carbides, in addition to the presence of C and Fe. Further analysis by high-resolution TEM (HRTEM) and the corresponding fast Fourier transform (FFT) pattern indicates that the carbide possesses an Fe_3_C-type structure. Therefore, we conclude that the carbide in 38CrMoAl steel is (Fe, Cr)_3_C.

**Figure 8:**
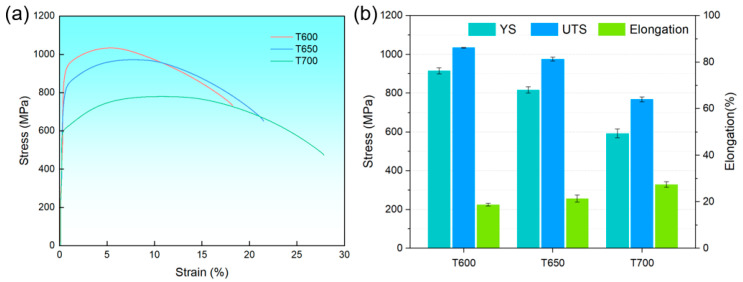
(**a**) Tensile curves and (**b**) mechanical properties of the T600, T650 and T700 samples. YS denotes yield strength; UTS denotes ultimate tensile strength.

Figure 8 illustrates the typical engineering stress–strain curves and mechanical properties of 38CrMoAl steel subjected to different tempering temperatures. When tempered at 600 °C, the steel exhibits the highest strength, with a yield strength (YS) of 915 ± 16 MPa and an ultimate tensile strength (UTS) of 1033.6 ± 1.6 MPa, yet it shows the lowest elongation of 18.7 ± 0.6%. As the tempering temperature increases to 650 °C, the YS decreases to 815 ± 16 MPa and the UTS drops to 974 ± 10 MPa, while the elongation rises to 21.3 ± 1.5%. A further increase in tempering temperature to 700 °C leads to a continued decline in strength, with YS and UTS reducing to 592 ± 22 MPa and 767 ± 12 MPa, respectively, accompanied by an increase in elongation to 27.3 ± 1.2%. Although the total elongation values under all three tempering conditions are relatively high, the uniform elongation remains considerably low. Specifically, the highest value of uniform elongation observed is only 10.3 ± 0.4%, which occurs at 700 °C, compared to 5.56 ± 0.14% at 600 °C. These results indicate that the 38CrMoAl steel possesses high strength but limited plasticity and work-hardening capacity.

### 3.2. The Influence of USSR Processing on the Microstructure, Mechanical Properties and Wear Resistance

Prior to USSR treatment, the surfaces of the samples were sequentially ground using sandpaper up to 800 grit. After grinding, the surface morphology of all samples appeared nearly identical, exhibiting a characteristic surface roughness (Ra) of 0.08 μm. Following USSR processing, the surface profiles of the three samples exhibited no marked variations, while the surface roughness increased to Ra 0.17 μm. This level of roughness is generally adequate for most engineering applications.

**Figure 9:**
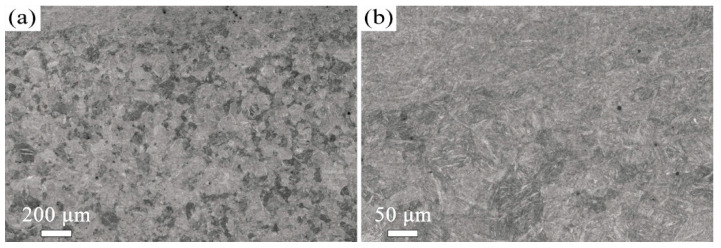
OM micrographs of the T600-USSR sample: (**a**) Low- and (**b**) high-magnification images.

**Figure 10:**
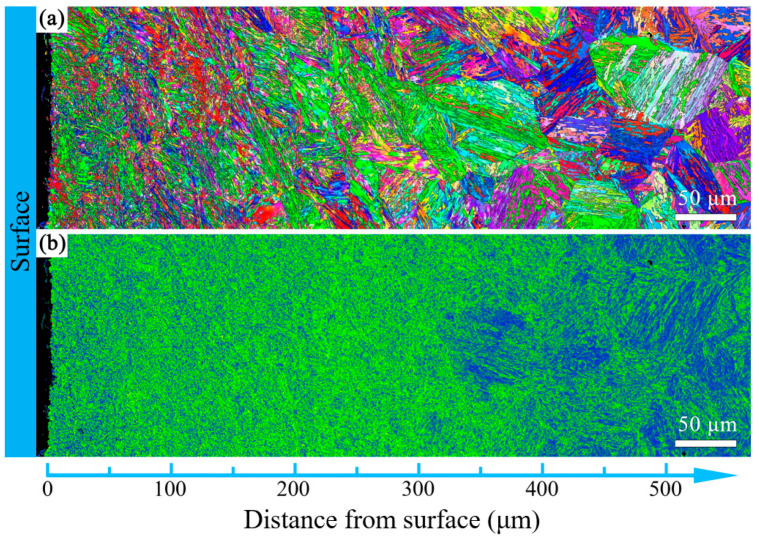
EBSD results of the T600-USSR sample: (**a**) IPF mapping and (**b**) KAM mapping.

**Figure 11:**
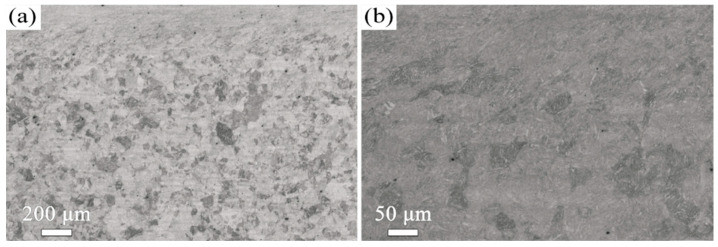
OM micrographs of the T650-USSR sample: (**a**) Low- and (**b**) high-magnification images.

**Figure 12:**
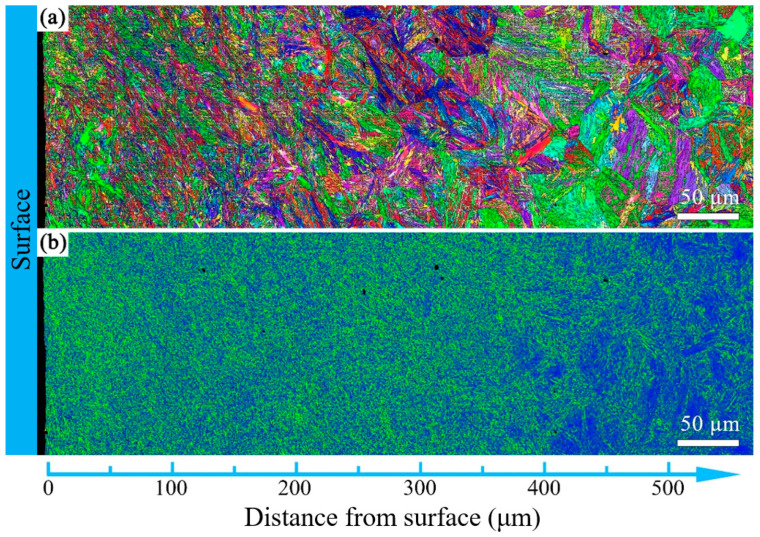
EBSD results of the T650-USSR sample: (**a**) IPF mapping and (**b**) KAM mapping.

**Figure 13:**
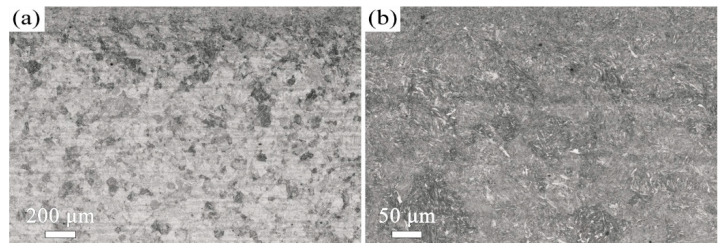
OM micrographs of the T700-USSR sample: (**a**) Low- and (**b**) high-magnification images.

**Figure 14:**
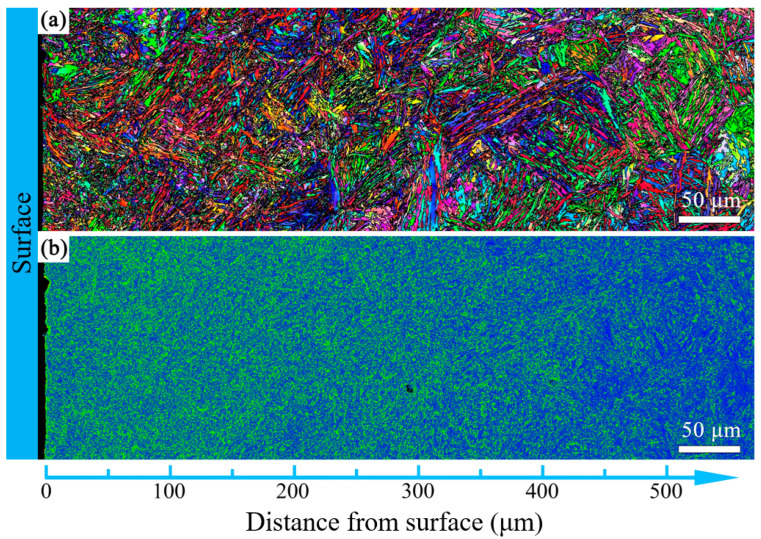
EBSD results of the T700-USSR sample: (**a**) IPF mapping and (**b**) KAM mapping.

The microstructure evolution with depth for the T600-USSR, T650-USSR, and T700-USSR samples is characterized through cross-sectional OM and EBSD, with the results presented in Figure 9, Figure 10, Figure 11, Figure 12, Figure 13 and Figure 14. Following USSR processing, the surface layers of all three samples exhibit severe plastic deformation along the rolling direction, elongating the primary austenite morphology without introducing microcracks. Furthermore, the microstructure in the topmost surface layer is significantly refined so that the original austenite and martensite morphologies become indistinguishable. This result indicates the formation of the gradient nanostructure.

**Figure 15:**
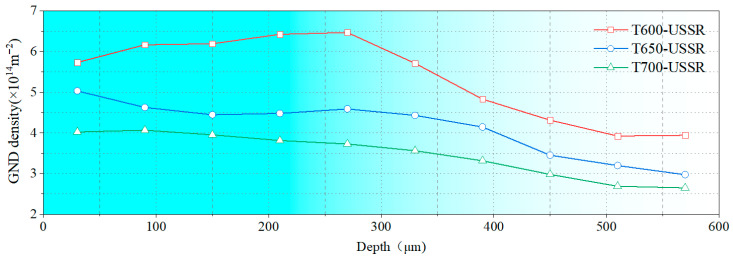
GND density variation with depth.

To quantify the gradient nanostructure, the EBSD data are divided into sub-regions by depth, and the GND density is calculated from KAM value. As shown in Figure 15, the GND density of all three samples demonstrates a gradient distribution. This confirms that USSR generates a defect-free gradient nanostructured surface layer, where gradients in grain size and dislocation density accommodate the plastic strain. In comparison, a higher tempering temperature results in a finer grain size and a higher GND density in the surface layer. This inverse relationship suggests that the efficacy of the USSR processing in refining the microstructure diminishes with an increase in the tempering temperature.

**Figure 16:**
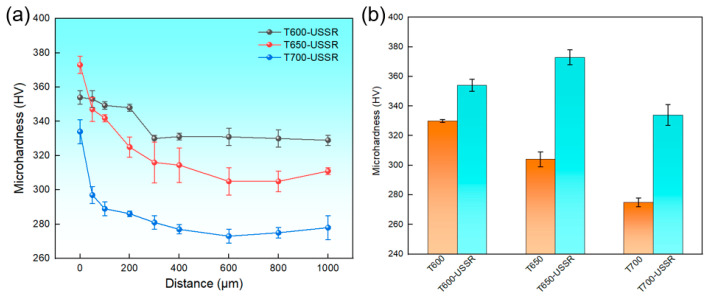
(**a**) Microhardness variation with the depth and (**b**) surface microhardness.

Figure 16a presents the variation in the microhardness as a function of depth for the three samples subjected to USSR processing. The results indicate a consistent decrease in hardness with increasing depth for all samples. This trend can be rationalized by the Hall–Petch relationship, which describes the inverse proportionality between strength and the square root of the grain size. Given that hardness is generally proportional to strength, the gradient nanostructure induced by USSR in 38CrMoAl steel results in a corresponding reduction in hardness. The gradient distribution of GND density also contributes to the gradient hardness profile through dislocation strengthening. Moreover, as the tempering temperature increases, the overall hardness profile shifts downward. In comparison, the T600-USSR sample exhibits a less pronounced hardness gradient, characterized by a smaller difference between its surface hardness and matrix hardness. Furthermore, with increasing tempering temperature, the surface hardness of the unprocessed sample gradually decreases. After USSR processing, the surface hardness is significantly enhanced to 354 ± 4 HV, 373 ± 5 HV, and 334 ± 7 HV, representing improvements of 7.3%, 22.7%, and 21.5%, respectively. A thickness increase is observed in the gradient nanostructured layer when the tempering temperature is raised, with measured values of 300 μm for the T600-USSR sample and 400 μm for the T700-USSR sample.

**Figure 17:**
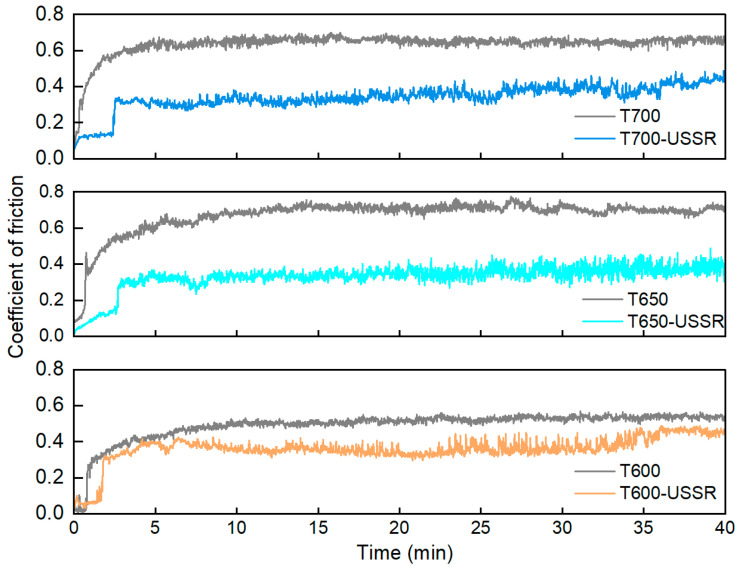
Variation of Coefficient of friction with sliding time.

**Figure 18:**
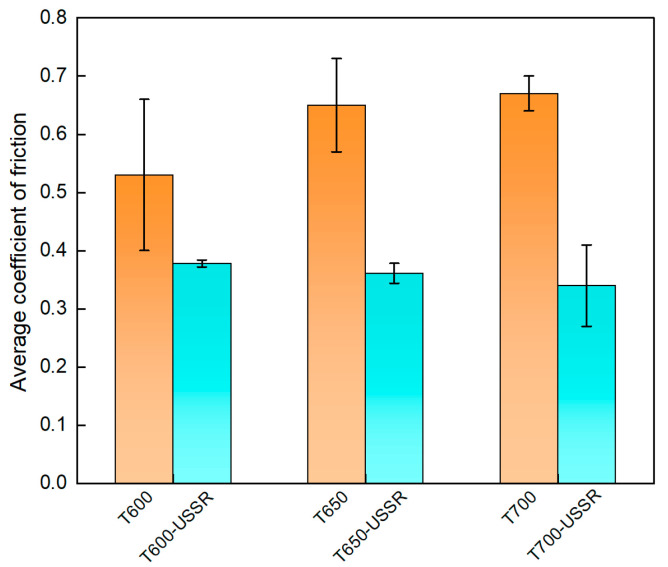
Average coefficient of friction.

Figure 17 depicts the evolution of the coefficient of friction as a function of sliding time, and Figure 18 presents the corresponding average value during the steady-state stage. The result indicates a notable reduction in the coefficient of friction following USSR processing across all samples. This decrease is primarily ascribed to the gradient nanostructure induced by the USSR processing, which enhances surface hardness and thereby improves resistance to deformation during wear. However, no statistically significant differences in the coefficient of friction are observed among the three USSR-processed samples.

**Figure 19:**
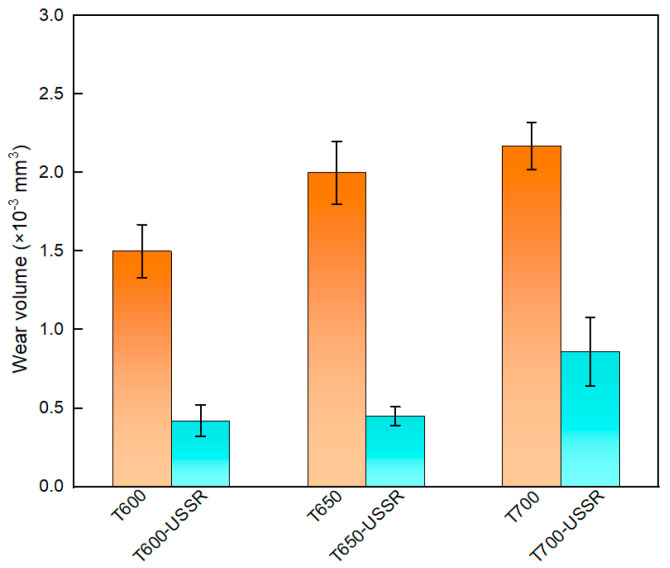
Wear volume.

The wear resistance of 38CrMoAl steel following USSR is further evaluated, and the result is shown in Figure 19. The results demonstrate a significant reduction in wear volume after USSR processing, indicating a notable improvement in wear resistance. The average wear volumes of the T600, T650, and T700 samples are 1.50 ± 0.17 × 10^−3^ mm^3^, 2.0 ± 0.2 × 10^−3^ mm^3^, and 2.17 ± 0.15 × 10^−3^ mm^3^, respectively, showing an increasing trend with higher tempering temperature. In contrast, the T600-USSR, T650-USSR, and T700-USSR samples exhibit average wear volumes of 0.4 ± 0.1 × 10^−3^ mm^3^, 0.45 ± 0.06 × 10^−3^ mm^3^. These values correspond to substantial wear volume reductions of approximately 73%, 78%, and 60% compared to their unprocessed counterparts. The most pronounced improvement is observed in the sample tempered at 650 °C, which achieves the highest reduction in wear volume. The superior wear resistance observed in the USSR-processed samples originates mainly from improved surface strength and hardness, resulting in a greater capacity to resist deformation.

## 4. Discussion

Based on our microstructural analysis, it is revealed that after USSR processing, the lath-like martensitic morphology disappears and is replaced by equiaxed fine grains in the near-surface region. This suggests that the USSR processing facilitates significant microstructure refinement, and even formation of nanograins at the topmost surface. Wang Et Al. reported that the refinement of lath martensite induced by LSP was achieved through dislocation accumulation, formation of DDWs (dense dislocation walls), and subsequent development of nanocrystals Via the sharpening of these DDWs [29]. Our previous study has also demonstrated that ferrite grain refinement induced by USSR processing occurs through a similar mechanism involving dislocation accumulation and the formation of DDWs [30]. The martensite phase exhibits an inherent lath structure, where the individual laths are only a few hundred nanometers wide and have slight misorientations with their neighbors [31]. Consequently, refinement can be achieved by segmenting these nanoscale laths through DDWs. Hence, the more pronounced grain refinement observed in the T600-USSR and T650-USSR tempered samples is attributed to their tempered martensite structure. In contrast, the T700 sample, composed of coarsened ferrite and carbides, exhibited less significant refinement after USSR processing. This difference accounts for the development of a more distinct gradient nanostructure in the T600-USSR and T650-USSR samples, as shown in Figure 9, Figure 10, Figure 11, Figure 12, Figure 13 and Figure 14.

The high strength of lath martensite is primarily derived from boundary strengthen, solid solution strengthening by supersaturated carbon atoms and its high dislocation density. However, elevated tempering temperatures promote a greater degree of martensite decomposition, which involves the precipitation of supersaturated carbon atoms as carbides and a concomitant reduction in dislocation density. This has been confirmed by our microstructural characterization, as shown in Figure 1, Figure 2, Figure 3, Figure 4, Figure 5, Figure 6 and Figure 7. Our analysis demonstrates that USSR introduces a high gradient strain, leading to the formation of a gradient grain structure and an increase in dislocation density within the surface layer. Consequently, the improvement in hardness arises primarily from refinement strengthening and dislocation strengthening mechanisms. It is also noted that higher tempering temperatures result in an overall lower hardness after USSR. For the T700-USSR sample, the maximum hardness is only comparable to the hardness of the T600 sample. In the case of the T650-USSR sample, only a surface layer approximately 170 µm thick exhibits hardness exceeding that of the T600 sample. Our EBSD analysis further reveals that the GND density in the near-surface region of the T700-USSR sample remains lower than the matrix GND density of the T600 sample. This observation leads us to conclude that the strengthening effects induced by USSR, namely grain refinement and dislocation strengthening, are insufficient to counteract the weakening caused by the extensive martensite decomposition at high tempering temperatures. Thus, a more optimal combination of properties for 38CrMoAl steel is achieved by combining tempering at 600 or 650 °C with the subsequent USSR process.

Our results indicate that the hardening effect induced by USSR is less pronounced in the low-temperature tempered sample. The limited enhancement observed in the T600 tempered sample can be primarily attributed to its low ductility and strain hardening capacity, as illustrated in Figure 8. In our previous study, it was demonstrated that USSR processing increased the surface hardness of 316L stainless steel from 237.83 HV to 442.27 HV, corresponding to an improvement of 204.44 HV [32]. The USSR resulted in a surface hardness increase of 72 HV in the brass [33]. This significant increase is largely due to the excellent ductility and strain hardening capacity of 316L stainless steel and brass. These findings lead us to conclude that surface severe plastic deformation techniques, such as USSR, may have limited effectiveness in enhancing the hardness of high-strength and low ductility metallic materials. Nonetheless, the gradient nanostructured surface layer induced by USSR paves the way for low-temperature nitriding—a process of considerable industrial interest—to further enhance the surface properties of 38CrMoAl steel, which will be the focus of our subsequent investigation.

## 5. Conclusions

In this study, to fabricate a defect-free gradient nanostructured surface layer on high-strength 38CrMoAl steel without compromising surface quality, the strength and ductility of quenched-and-tempered samples are first adjusted by tempering at 600 °C, 650 °C, and 700 °C, followed by USSR processing. The influence of the resulting gradient nanostructure on mechanical properties and wear resistance is systematically investigated, along with the underlying mechanisms. The main conclusions are as follows:

(1) The microstructure of the 38CrMoAl steel evolves from tempered martensite with fine carbides at 600 °C to a ferrite matrix with coarse carbides at 700 °C. This microstructural transition leads to a decrease in yield strength (from 915 ± 16 MPa to 815 ± 16 MPa) but an increase in elongation (from 18.7 ± 0.6 to 27.3 ± 1.2%). An excellent combination of strength and ductility is achieved in the samples tempered at 600 °C and 650 °C.
(2) Following USSR processing, all tempered samples develop a defect-free gradient nanostructured surface layer, evidenced by progressively coarser martensite/ferrite grains and reduced dislocation density with depth, alongside a smooth surface (Ra 0.17 μm). The thickness of this layer expands with increasing tempering temperature, from 300 μm at 600 °C to 400 μm at 700 °C.
(3) After USSR processing, the samples tempered at 600 °C, 650 °C, and 700 °C exhibit a notable increase in surface hardness, reaching 354 ± 4 HV, 373 ± 5 HV, and 334 ± 7 HV, respectively—corresponding to improvements of 7.3%, 22.7%, and 21.5% over their unprocessed counterparts. Their wear volume decreases significantly by approximately 73%, 78%, and 60% for each tempering condition, respectively. Notably, the wear volumes of the samples tempered at 600 °C and 650 °C are comparable and substantially lower than that of the sample tempered at 700 °C. USSR treatment also leads to a reduction in the coefficient of friction.

The results collectively indicate that the USSR-processed samples tempered at 600 °C and 650 °C possess good mechanical properties, coupled with outstanding wear resistance. Although both perform exceptionally, the 650 °C condition is recommended for applications requiring superior ductility, while the 600 °C condition may be chosen for scenarios where slightly higher strength is critical.

## Data Availability

The original contributions presented in this study are included in the article. Further inquiries can be directed to the corresponding authors.
